# Whole genome sequence of the deep-sea sponge *Geodia barretti* (Metazoa, Porifera, Demospongiae)

**DOI:** 10.1093/g3journal/jkad192

**Published:** 2023-08-24

**Authors:** Karin Steffen, Estelle Proux-Wéra, Lucile Soler, Allison Churcher, John Sundh, Paco Cárdenas

**Affiliations:** Pharmacognosy, Department of Pharmaceutical Biosciences, Uppsala University, Uppsala 751 24, Sweden; Department of Biochemistry and Biophysics, National Bioinformatics Infrastructure Sweden, Science for Life Laboratory, Stockholm University, Solna SE-17121, Sweden; Department of Medical Biochemistry and Microbiology, National Bioinformatics Infrastructure Sweden (NBIS), Science for Life Laboratory, Uppsala University, Uppsala 752 37, Sweden; Department of Molecular Biology, National Bioinformatics Infrastructure Sweden, Science for Life Laboratory, Umeå University, Umeå 901 87, Sweden; Department of Biochemistry and Biophysics, National Bioinformatics Infrastructure Sweden, Science for Life Laboratory, Stockholm University, Solna SE-17121, Sweden; Pharmacognosy, Department of Pharmaceutical Biosciences, Uppsala University, Uppsala 751 24, Sweden

**Keywords:** *Geodia barretti*, Porifera, Tetractinellida, Sweden, symbionts, metagenome-assembled genome

## Abstract

Sponges are among the earliest branching extant animals. As such, genetic data from this group are valuable for understanding the evolution of various traits and processes in other animals. However, like many marine organisms, they are notoriously difficult to sequence, and hence, genomic data are scarce. Here, we present the draft genome assembly for the North Atlantic deep-sea high microbial abundance species *Geodia barretti* Bowerbank 1858, from a single individual collected on the West Coast of Sweden. The nuclear genome assembly has 4,535 scaffolds, an N50 of 48,447 bp and a total length of 144 Mb; the mitochondrial genome is 17,996 bp long. BUSCO completeness was 71.5%. The genome was annotated using a combination of ab initio and evidence-based methods finding 31,884 protein-coding genes.

## Introduction

Sponges (phylum Porifera) hold an evolutionarily important position as one of the earliest animal lineages (Redmond and McLysaght 2021; Schultz *et al*. 2023). Sponges do not produce organs but feature instances of true epithelial tissue, a hallmark of metazoans (Leys and Riesgo 2012). Their body features cells of varying complexity and fate (Musser *et al*. 2021), and only recently, major breakthroughs in sponge cell cultures were made (Conkling *et al*. 2019). Among the focal aspects of research on sponges are their remarkable microbiota and chemical diversities (Thomas *et al*. 2016; Calado *et al*. 2022).

**Fig. 1.**
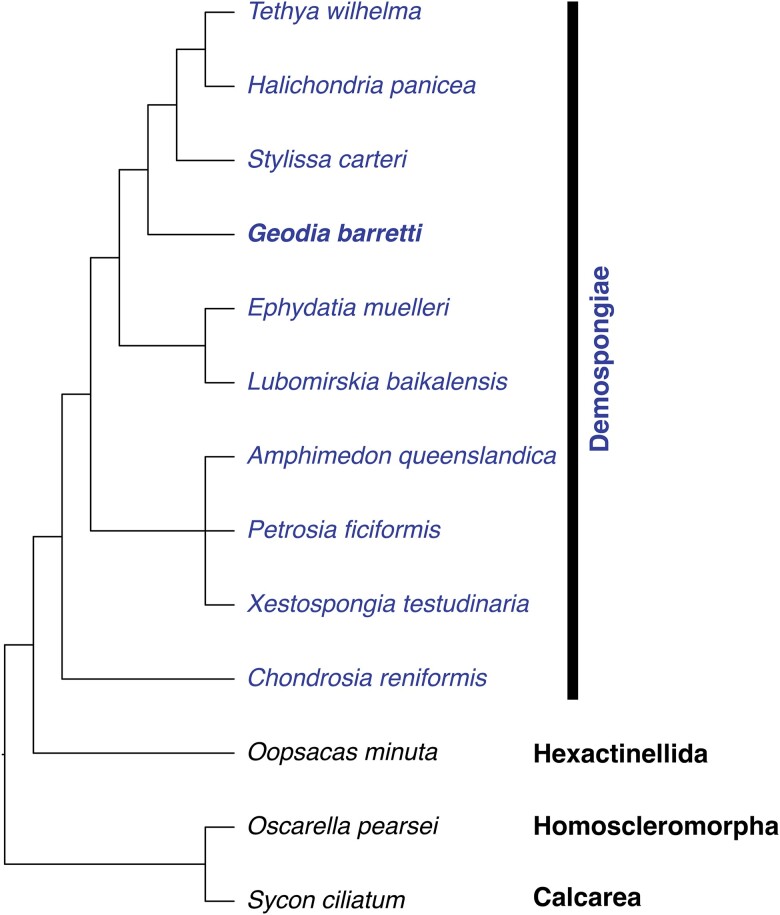
Cladogram of all sponge species with genomes published to date, drawn after (Morrow and Cárdenas 2015; Plese *et al*. 2021).

In the past, these 2 aspects complicated access to their genomes, as DNA extracted from sponges is contaminated by microbial DNA and by compounds binding DNA (Marshall and Barrows 2004) and potentially interfering with sequencing. Workarounds like producing a genome from DNA of thousands of larvae which naturally have a lower abundance of microbial symbionts (Srivastava *et al*. 2010) or whole-genome amplification (WGA) from single cells (Ryu *et al*. 2016) both yielded highly fragmented assemblies. Recent sequencing strategies (long reads, synthetic long reads, and/or Hi-C) yield chromosome-level assemblies, as in the case of *Ephydatia muelleri* (Kenny *et al*. 2020) as well as *Petrosia ficiformis* and *Chondrosia reniformis* (McKenna *et al*. 2021). However, 13 years after the first sponge genome, there were only 12 sponge genomes available, of over 9,500 described species of sponges (Fig. 1; Table 1) (de Voogd *et al*. 2023).

**Table 1.** Sponge genomes and their respective repositories.

Species	Genome repository
*A. queenslandica* (1) (Srivastava *et al*. 2010)	https://www.ncbi.nlm.nih.gov/assembly/GCF_000090795.2
*A. queenslandica* (2)	https://www.ncbi.nlm.nih.gov/data-hub/genome/GCA_016292275.1/
*E. muelleri* (Kenny *et al*. 2020)	https://www.ncbi.nlm.nih.gov/assembly/GCA_013339895.1
*H. panicea* (Strehlow *et al*. 2022)	https://www.ncbi.nlm.nih.gov/assembly/GCA_020423275.1
*O. minuta* (Santini *et al*. 2023)	https://www.ncbi.nlm.nih.gov/assembly/GCA_024704765.1
*P. ficiformis*	https://www.ncbi.nlm.nih.gov/assembly/GCA_947044365.1
*C. reniformis*	https://www.ncbi.nlm.nih.gov/assembly/GCA_947172415.1
*S. carteri* (Ryu *et al*. 2016)	http://sc.reefgenomics.org/download/
*X. testudinaria* (Ryu *et al*. 2016)	http://xt.reefgenomics.org/download/
*T. wilhelma* (Francis *et al*. 2017)	https://bitbucket.org/molpalmuc/tethya_wilhelma-genome/src/master/
*L. baikalensis* (SPAdes_500 min.fa) (Kenny *et al*. 2019)	https://figshare.com/articles/dataset/Transcriptomic_and_genomic_assemblies_Lake_Baikal_Sponge_Data/6819812
*O. pearsei* (Nichols *et al*. 2012)	https://figshare.com/articles/dataset/Oscarella_pearsei_assemblies/7107638
*S. ciliatum* (Fortunato *et al*. 2012)	https://datadryad.org/stash/dataset/doi:10.5061/dryad.tn0f3

**Fig. 2.**
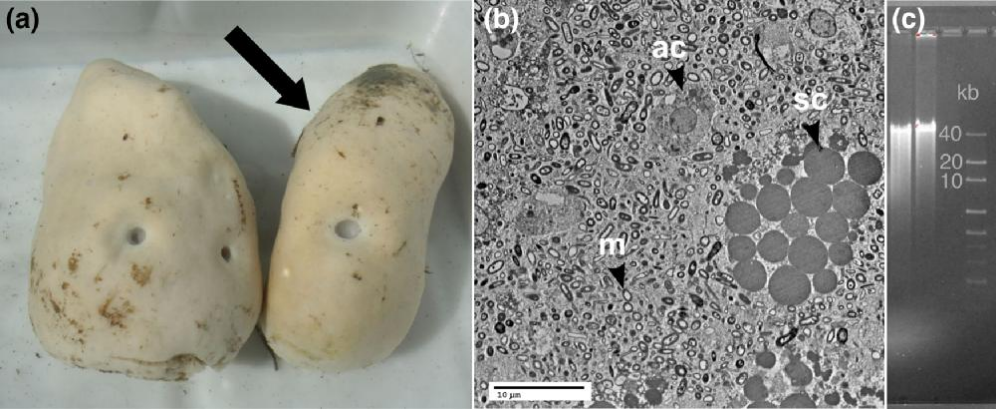
a) *G. barretti* specimens freshly collected on 4 May 2016, Kosterfjord, Sweden: left (UPSZMC 184976) and right (UPSZMC 184975) with arrow, the specimen chosen for whole-genome sequencing. b) TEM image of *G. barretti* (UPSZMC 184975) showing 2 types of sponge archeocyte cells archaeocytes (ac) and spherulous cells (sc). All other smaller bodies are microbial symbionts (m). Image courtesy of V. Koutsouveli and A. Riesgo, NHM London. c) DGGE image of DNA prior to PacBio library production. Image courtesy of SciLifeLab Uppsala.

*Geodia barretti* Bowerbank 1858 (Fig. 2a) is a widespread North Atlantic deep-sea demosponge found in depths of 30–2,000 m (Cárdenas *et al*. 2013) and, thus, would represent 1 of the few genomes of a deep-sea animal. As a high microbial abundance (HMA) sponge, *G. barretti* hosts an outstanding density and diversity of microbes (Fig. 2b) with an average of 3 × 10^10^ microbes/cm^3^ (Hoffmann *et al*. 2006; Leys *et al*. 2018) from over 400 prokaryotic amplicon sequence variants across 17 phyla (Radax *et al*. 2012; Steffen *et al*. 2022). The characterization as “HMA” (highly abundant and diverse microbiota) and “LMA” (lowly abundant and species-poor microbiota) sponges has been recognized since the 1970s and is a species specific attribute, but its significance to the organisms is not yet fully elucidated (Vacelet 1975; Hentschel *et al*. 2003). This microbiota partly accounts for its richness in natural products (Erngren *et al*. 2021; Steffen *et al*. 2021) with still many unknown bioactive metabolites. Finally, *G. barretti* is a key species of sponge grounds, deep-sea habitats characterized by mass accumulation of sponges (Klitgaard and Tendal 2004; Cárdenas *et al*. 2013). Sponge grounds are considered vulnerable marine ecosystems (VMEs) and as such *G. barretti* is part of the VME indicator species list (ICES 2019). Its physiology has been extensively studied to understand the many ecosystem services it provides (Cárdenas and Rapp 2013; Koutsouveli, Cárdenas, Santodomingo, *et al*. 2020; Maier *et al*. 2020; Rooks *et al*. 2020; Bart *et al*. 2021) as well as to investigate its resilience to human activities (Kutti *et al*. 2015; Colaço *et al*. 2022) including climate change (Strand *et al*. 2017). Therefore, producing a genomic resource for this species is not only valuable for conservation efforts but also for a more general understanding of deep-sea benthic ecosystems.

## Methods and materials

### Sampling

The specimen of *G. barretti* (Tetractinellida, Astrophorina, Geodiidae) (Fig. 2a) was collected on 4 May 2016 with a basket fixed to an ROV on board the R/V *Nereus* in the Kosterfjord National Park, Sweden, West of Yttre Vattenholmen (58.876233, 11.101483) at 96-m depth. The sample was identified on board by P. Cárdenas. Small-tissue sections were immediately flash-frozen in liquid nitrogen, while larger pieces were frozen at −20°C, and the rest was kept as a voucher and fixed in ethanol 96% (ethanol changed twice). The voucher is stored in 96% ethanol at the Museum of Evolution, Uppsala, Sweden, under museum number UPSZMC 184975. *G. barretti* is a gonochoric species, but the sex of the specimen could not be determined since it was not reproducing at the time of its collection and did not contain any observable larvae as it is oviparous (= “specimen NR_1” in Koutsouveli, Cárdenas, Conjeco, *et al*. 2020). During this same reproduction study, transmission electron microscope (TEM) pictures were made from UPSZMC 184975, confirming the high abundance of microbes (Fig. 2b).

### DNA extraction and sequencing

DNA extraction for whole-genome sequencing was impeded by rapidly degrading DNA and chemical contamination coisolated with the DNA. We obtained the best results by macerating flash-frozen tissue in 0.2 M EDTA pH 8.0 and straining the dissociated cells through a filter (40 µm, Nalgene) before extracting DNA using a traditional chloroform/isoamyl alcohol partitioning protocol (Dharamshi *et al*. 2022). The resulting DNA was assayed by NanoDrop (passing range: 260/280, 1.8–2.2; 260/230, 2.0–2.2) and denaturing gradient gel electrophoresis (Fig. 2c), and suitable fractions were sequenced on PacBio (RSII and Sequel) and Illumina (HiSeqX) platforms, all performed by the SNP&SEQ Technology Platform, SciLifeLab Uppsala, Sweden.

### Data processing

#### RNAseq data preparation

Poly-A selected RNA-sequencing (RNAseq) data of UPSZMC 184975 and 6 other *G. barretti* individuals were used for identification of sponge contigs and gene annotation (Koutsouveli, Cárdenas, Santodomingo, *et al*. 2020). For identification of sponge contigs in the subsequent whole-genome assembly, the RNAseq data were de novo assembled using Trinity (Haas *et al*. 2013) with default parameters. For annotation, the same RNAseq data were reassembled with fastp (Chen *et al*. 2018), hisat2 (Kim *et al*. 2015), and StringTie (Pertea *et al*. 2015) with the genome assembly in order to avoid a high number of small unsupported genes and erroneous transcripts.

### Assembly

PacBio data were assembled with Flye using the “-meta” flag (Kolmogorov *et al*. 2020) and polished with the Illumina short reads using one round of Pilon (Walker *et al*. 2014).

To remove contamination (i.e. nonsponge contigs/scaffolds) and only keep sponge contigs, 2 different strategies were combined: taxonomic identification of contigs and contig coverages with RNAseq data. First, taxonomic classification of the contigs was performed by both contigtax (https://github.com/NBISweden/contigtax, v0.5.9) and BlobTools (Laetsch and Blaxter 2017). In order to run the latter, the short reads were aligned to the assembly with BWA (Li and Durbin 2009); and using the assembled contigs, we performed both a blastn search (Altschul *et al*. 1990) against the nt database and a DIAMOND blastx search (Buchfink *et al*. 2015) against the nr database. The obtained BAM file and BLAST output files were used as input files for BlobTools. We kept all contigs annotated as “Eukaryota” by both contigax and BlobTools as a first noncontaminated set for the sponge assembly. Second, 3 of the de novo built transcriptomes were mapped on the full assembly using gmap (Wu *et al.* 2016). The contigs that on average for the 3 transcriptomes had at least 20% of their length mapped by transcripts were also added to the noncontaminated set of contigs. Coverage was calculated by mapping short and long reads to the genome with BWA and minimap2 (Li 2018), respectively. The coverage per position was extracted from the resulting BAM file using samtools depth (Li *et al*. 2009) and the average and median across all positions was calculated.

### Annotation

All parts of the annotation workflow (annotation preprocessing, transcript assembly, ab initio training, and functional annotation) were performed with 4 Nextflow pipelines from https://github.com/NBISweden/pipelines-nextflow. For annotation preprocessing, all nucleotides were changed to uppercase to prevent interpretation as repeats. The Ns at start or end of contigs are trimmed out to avoid problems when submitting data to public archives. Repeats were masked using RepeatModeler package. Candidate repeats modeled by RepeatModeler were vetted against our protein set (minus transposons) to exclude any nucleotide motif stemming from low-complexity coding sequences. From the repeat library, identification of repeat sequences present in the genome was performed using RepeatMasker (Smit *et al*. 2013) and RepeatRunner (Smith *et al*. 2007).

The genome was annotated using the MAKER package (Holt and Yandell 2011), and both evidence-based (using the transcriptomes and gene sets) and ab initio approaches (optimize_augustus.pl) were performed in several (3) iterations until the number of false positive predictions clearly decreased. The annotation quality is given by the annotation edit distance (AED) provided in the gff file. The functional annotation was performed with an in-house pipeline (https://github.com/NBISweden/pipelines-nextflow/tree/master/subworkflows/functional_annotation) based on BLAST and InterProScan. tRNAs were annotated by tRNAscan, and only those with an AED < 1 are reported. The mtDNA was recovered in a single contig and annotated by lifting over annotations from the mtDNA of *Geodia neptuni* (AY320032) with Geneious v 8.1.9 at a similarity threshold of 75%; ORFs were annotated using genetic code 4. The annotations were adapted to EMBL format using a gff conversion tool (Norling *et al*. 2018).

### Comparative genomics

For placing this sponge genome in its context, all other available sponge genome assemblies known to us were downloaded from NCBI GenBank or their respective repositories (Table 1). After initial analysis, 6 assemblies were excluded. Six genomes of *Aplysina aerophoba* under BioProject PRJEB24804 appear uncharacteristically small (largest assembly is 3 Mb) and mainly consist of microbial symbiont sequences (D. Sipkema, *pers. comm*.). In addition, the alternate pseudohaplotype of *P. ficiformis* (GCA_947044245.1) was excluded as it is only 12% complete according to BUSCO, as was *C. reniformis* (GCA_947172445.1). For *Amphimedon queenslandica*, there are currently 3 genome assemblies available: the original first sponge genome “v1.0” (GCA_000090795.1) (Srivastava *et al*. 2010), superseded by a second version “v1.1” (GCA_000090795.2), followed by a third assembly from the same research group “UQ_AmQuee_3” (GCA_016292275.1) but from a different/unrelated specimen and sequencing project. Although there is no publication for this third genome assembly, it was included here since it seemed highly complete.

Genome completeness was assessed with BUSCO and metazoa_odb10 (Simão *et al*. 2015). For identification of biosynthetic gene clusters (BGCs), genomes were vetted by antiSMASH (bacterial version) using prodigal-m as gene finder with default parameters (Blin *et al*. 2021). All figures were created in R (R Core Team 2016) using the packages within tidyverse.

## Reproducibility

### GitHub repositories and versions

AGAT repository: agat 0.6.2, commit 338be8; GAAS repository: gaas 1.2.0, commit 9af467.

Nextflow pipeline repository: commit 612364.

### Tool versions

Flye (2.4.2), Pilon (1.22), BUSCO (5.2.2/5.3.1), gmap (2018-02-12), Trinity (2.11.0), antiSMASH (5.2.1), BWA (0.7.8, 0.7.17), contigtax (v 0.5.9) with UniRef90 database (v2019_11), BlobTools (v1.1.1), minimap2 (2.4), samtools (1.9), fastp (0.20.0), hisat2 (2.1.0), StringTie (2.0), RepeatModeler package (1.0.11), RepeatMasker (4.0.9_p2), RepeatRunner, MAKER package (3.01.02), exonerate (2.4.0), BLAST (2.9.0), Bioperl (1.7.2), Augustus (3.3.3), TRNAscan-se (1.3.1), Snap (version 2013_11_29), GeneMark-ET (4.3), GeneMark (ES Suite version 4.48_3.60_lic), InterProScan (5.30–69.0), Infernal (1.1.2), Prokka (1.11), and R (v4.2.1) using tidyverse (1.3.1) packages for visualization.

### Databases versions

UniProt Swiss-Prot database (downloaded on 2020-12; 563 972 proteins), Rfam version 14.4.

## Results and discussion

### The *G. barretti* genome assembly

We generated 2,364,732 (2.4 M) reads with long-read technologies (PacBio RSII, Sequel) for an average coverage of 18.82632 (19×, median 14×) and 427,393,248 (427.4 M) reads with short-read technology (Illumina HiSeqX) for an average coverage of 339.9417 (340×, median 256). Sponge DNA degraded in PacBio library production and hence long-read data output was low. The reason for this breakdown of sponge DNA is currently unknown.

Assessing the initial metagenomic assembly from BlobTools results, at the super kingdom level, the reads mapping the metagenome assembly were classified as Eukaryota (37.2%) and bacteria (37.2%). 20.5% of the mapped reads were annotated as “no-hit.” At the phylum level, the mapped reads were mostly classified as Porifera (28.3%), “no-hit” (20.5%), “other” (16.3%), Proteobacteria (15.3%), and Chordata (4.3%) (Supplementary Figs. 1 and 2); 6.9% of the mapped reads were annotated as *Candidatus* Poribacteria, one of the most abundant microorganisms in demosponges (Lafi *et al*. 2009) including in *G. barretti* (Radax *et al*. 2012; Steffen *et al*. 2022), although its abundance is often greatly underestimated due to 16S rRNA primer biases (Steinert *et al*. 2017; Steffen *et al*. 2022). In total, the mapped reads covered 66 phyla, showing the complexity of microbial communities residing within marine sponges. Similar results are observed with contigtax. At the super kingdom level, more than half of the contigs were annotated as “unclassified,” a third as bacteria, and only 12% as Eukaryota. Among the contigs annotated as bacteria, the majority (30%) belong to the phylum *Candidatus* Poribacteria. Other major categories are Proteobacteria (19%) and Chloroflexi (14%), 2 phyla that have been identified as frequent and abundant symbionts in sponge microbiota (Thomas *et al*. 2016) including in *G. barretti* (Radax *et al*. 2012; Steinert *et al*. 2017; Steffen *et al*. 2022). Among the eukaryotic contigs, 72% are annotated as Porifera. For comparison, Supplementary Figs. 3 and 4 contain the final assembly evaluated with BlobTools showing a decrease in contribution of foreign sequences to the genome assembly.

The genome assembly has a length of 144,789,364 bp (144.7 Mb) across 4,535 contigs. There are 110 Ns in the genome, and it has a GC content of 49.3%. According to BUSCO (v. 5.3.1 metazoa_odb10), the genome is 71.5% complete with 3.6% duplicates of single-copy orthologs. N50 length is 48,446 (contig size ranging from minimum 1,002 bp and median 22,605 bp to maximum 495,233 bp). For comparison, the haploid genome size was estimated to be 127 Mb based on a *C*-value of 0.13 pg measured by Feulgen image analysis densitometry (FIAD) (Ryan Gregory and Darren Kelly, *pers. comm.*). The excess sequences could be due to noncollapsed heterozygous regions in the sponge genome and/or incorporation of microbial symbiont sequences in the genome. As part of the genome assembly, we recovered the mitochondrial genome in 1 chromosome. The mtDNA was circular and had a length of 17,996 bp and the characteristic synteny of tetractinellid mtDNA (Plese *et al*. 2021): rnl-cox2-atp8-atp6-cox3-cob-atp9-nad4-nad6-nad3-nad4L-cox1-nad1-nad2-nad5-rrns. Beyond the sponge genome, the sequencing data are in fact metagenomic data, and we invite the use of it for exploration of the microbial and viral communities of *G. barretti* UPSZMC 184975, which was beyond the scope of our work.

In the assembly, RepeatMasker annotated 117,982 repeats with a total size of 26,945.75 kb or 18.61% of the genome (mean 228.47 bp). RepeatRunner annotated 1,043 repeats with a total size of 631.47 kb or 0.44% of the genome (mean 605.43 bp). The difference in results is to be expected as the 2 programs are complementary. RepeatMasker identifies repeats based on similarity to known repeats, whereas RepeatRunner identifies highly divergent repeats.

The genome annotation contains 31,884 protein-coding genes. The BUSCO scores together with the number of annotated protein-coding genes suggest that protein-coding genes are well represented in the assembled sequence. There were 66,936 mRNAs (as there were several isoforms per gene) with an average of 7.2 exons per mRNA, an average exon length of 244 bp, and an average coding sequence length of 1,122 bp. Of those genes, 27,544 were functionally annotated, as were 59,664 of the mRNAs. The genome contained 156 tRNAs with an AED < 1.

### Comparison with currently available sponge genomes

**Fig. 3.**
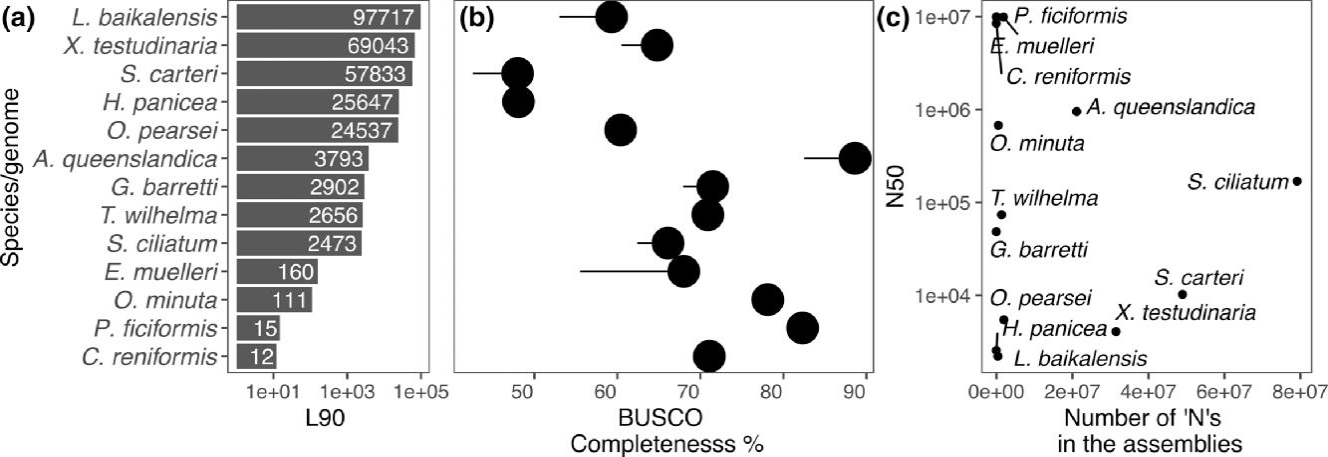
a) L90, number of contigs that cumulatively cover 90% of the assembly and b) the corresponding BUSCO completeness (C). The tail indicates the number of duplicate BUSCOs. c) The N50 compared to the number of Ns in the assemblies.

It is worthwhile noting that about half of the 12 previously published sponge genomes are not deposited in widely used databases such as NCBI GenBank or ENA but in other data repositories. Therefore, all currently valid download links are summarized in Table 1. To place our genome in its context, we summarized technical assembly metrics in Table 2 and biological metrics in Table 3 and visualize a subset in Fig. 3.

**Table 2.** Various genome assembly metrics.

				Contiguity				BUSCO scores				
Species	Shortest contig	Longest contig	Assembly size	contig number	count of N's	N(G)50	L90	C%	S%	D%	F%	M%
*A. queenslandica* (1)	633	1,888,931	164,262,607	13,133	21,126,302	123,180	3,793	88.6	82.5	6.1	3.9	7.5
*A. queenslandica* (2)	314	4,599,197	167,703,827	3,871	22,857,408	950,481	268	89.1	83.5	5.6	3.7	7.2
*C. reniformis*	6,754,398	10,413,042	117,372,766	14	5,600	8,459,200	12	71.1	70.4	0.6	12.2	16.8
*E. muelleri*	282	34,737,626	322,619,961	1,434	1,864,700	9,883,643	160	68	55.5	12.6	9.3	22.6
***G. barretti***	**1,002**	**495,233**	**144,789,364**	**4,535**	**110**	**48,447**	**2,902**	**71**.**5**	**67**.**9**	**3**.**6**	**12**.**6**	**15**.**9**
*H. panicea*	949	50,219	73,970,439	32,385	0	2,555	25,647	48.1	47.7	0.4	28.1	23.8
*L. baikalensis*	500	124,926	209,989,122	135,191	373,641	2,213	97,717	59.3	53	6.3	19.6	21.1
*O. minuta*	1002	1,926,057	61,460,524	365	501,094	676,369	111	78.1	77.3	0.8	4.6	17.3
*O. pearsei*	100	107,672	57,775,306	67,767	1,985,416	5,457	24,537	60.4	59.7	0.6	16	23.6
*P. ficiformis*	4,740,285	63,279,122	191,074,932	18	15,000	9,942,894	15	82.3	81.4	0.8	6.5	11.2
*S. carteri*	800	965,826	418,920,728	97,497	48,953,456	10,236	57,833	48	42.6	5.5	28.5	23.5
*S. ciliatum*	1,001	1,380,240	357,509,570	7,780	79,115,821	169,232	2,473	66.1	62.4	3.8	14.9	19
*T. wilhelma*	1,004	659,656	125,670,620	5,936	1,516,047	73,701	2,656	70.9	69.2	1.7	12.7	16.5
*X. testudinaria*	800	1,572,474	257,935,546	97,640	31,482,608	4,078	69,043	64.8	60.5	4.3	14.8	20.4

N(G)50, size of the smallest contig that, with all larger contigs, sums up to over half of the assembly length; L90, number of contigs to span 90% of the genome assembly; BUSCO, total BUSCOs searched are 954; values given in the table are the percentages of the total BUSCOs that were identified respectively in each genome. C, complete BUSCOs; S, complete and single-copy BUSCOs; D, complete and duplicated BUSCOs; F, fragmented BUSCOs; M, missing BUSCOs. *A. queenslandica* 1 is GCF_000090795.2; *A. queenslandica* 2 is GCA_016292275.1. Species listed are in alphabetical order. In bold the genome reported herein.

**Table 3.** Number of BGCs according to antiSMASH and biological classification (type) as “HMA” or “LMA” species.

Species	Number of BGCs	Type	GC%	Gene number/CDS	% repetitive
*A. queenslandica* (1)	1	LMA	35.5	30,327	43
*A. queenslandica* (2)	2	LMA	35.8	—	—
*C. reniformis*	1	HMA	37.3	—	—
*E. muelleri*	10	LMA	43.2	39,245	47
***G. barretti***	**21**	**HMA**	**49**.**3**	**31,844**	**19.05**
*H. panicea*	4	LMA	42.2	—	—
*L. baikalensis*	69	LMA	43.8	—	—
*O. minuta*	4	LMA	35.7	16,413	—
*O. pearsei*	3	LMA	43.5	9,823	—
*P. ficiformis*	3	HMA	33.9	—	—
*S. carteri*	15	LMA	43.9	26,967	—
*S. ciliatum*	9	LMA	47.0	—	—
*T. wilhelma*	3	LMA	39.9	37,416	—
*X. testudinaria*	133	HMA	49.9	22,337	—

GC % was calculated from individual nucleotide counts. The numbers of genes/CDS and the percentage of repetitive sequences were taken from the respective publications. Species are listed in alphabetical order. In bold the genome reported herein.

Assembly sizes in sponges range from 58 to 419 Mb (Table 2), which is in the range of genome sizes reported in the literature. Using FIAD and flow cytometry across a set of 75 sponge species, *Tethya actinia* and an unknown Dictyonellidae had the smallest genome with 39.1 Mb, while *Mycale laevis* 616.1–694.4 Mb and *Placospongia intermedia* 528.1–782.4 Mb had the largest genomes (Jeffery *et al*. 2013). In terms of difference between the size of genome assembly and genome size measured from cells, the assembly for *Xestospongia testudinaria* is almost 60% larger than the genome size estimated by flow cytometry (161.37 vs 258 Mb assembly). Indeed, both *X. testudinaria* and *Stylissa carteri* assemblies are hologenomes, and this excess sequence could indicate significant microbial contamination and/or high heterozygosity.

We selected a set of frequently used metrics to place the genome assembly of *G. barretti* in context with the other sponge genomes available (Table 2). The first set includes various metrics to express contiguity, i.e. the degree of fragmentation in the assembly. Ideally, the number of contigs should be the number of chromosomes (haploid), which is the case for the genomes of *C. reniformis* and *P. ficiformis*. The genome assembly of *E. muelleri* represents 23 chromosomes in 24 scaffolds but opted to also include a number of unplaced contigs, thus increasing the total number of contigs.

The second set of metrics in Table 2 was produced by BUSCO, a tool approximating biological completeness of a genome assembly by assessing the presence or absence of near universal single-copy genes (orthologs) (Simão *et al*. 2015). The numbers in this table may deviate from the values given in the original publications as there are pronounced differences between different versions of BUSCO and different reference gene sets. For comparability, we computed the metrics again, all with the same version of the program (v. 5.3.1, metazoa_odb_10, 2021-02-24). Overall completeness (C) frequently used to describe assemblies ranged from 48 to 89%. Generally, while higher values are better, some genes may truly be absent thus not allowing for a 100% completeness. Genes (BUSCOs) may also escape detection due to technical limitations, mainly due to gene prediction difficulties for highly derived lineages. The 2 chromosome-level assemblies for instance score “only” 71.1 and 82.3% complete. This “completeness” is the sum of single (S) and duplicate (D) BUSCOs identified. The number of duplicate BUSCOs is an important parameter as it can be indicative of whether diploid genomes were correctly and consistently collapsed to a haploid assembly, which can be an issue in organisms with high heterozygosity and/or when assembling long reads (Guiglielmoni *et al*. 2021). The highest number of duplicate single-copy orthologs (12.6%) was detected in *E. muelleri* assembly.

For the sponge genome assemblies published to date, different strategies were employed to isolate the starting material, DNA. Most of the genomes are “single origin” meaning that all DNA was extracted from a single individual: *C. reniformis*, *Halichondria panicea*, *Lubomirskia baikalensis*, *Oscarella pearsei*, *P. ficiformis*, *S. carteri*, *Tethya wilhelma* (D. Erpenbeck and W. Francis, *pers. comm.*), *X. testudinaria*, and *G. barretti*, the genome presented herein. However, several assemblies are based on DNA extracted from several (*Sycon ciliatum* (M. Adamska, *pers. comm.*), *Oopsacas minuta* (E. Renard, *pers. comm.*) to thousands of individuals (*A. queenslandica* (Srivastava *et al*. 2010; B. Degnan, *pers. comm.*). Most frequently, adult biomass (“tissue”) was extracted (*C. reniformis*, *H. panicea*, *L. baikalensis*, *O. minuta*, *P. ficiformis*, *S. carteri*, *S. ciliatum*, *T. wilhelma*, *X. testudinaria*, and *G. barretti*), but in some cases, whole larvae were extracted instead (*A. queenslandica* (Srivastava *et al*. 2010; B. Degnan, *pers. comm.*; *O. pearsei*). Notably, sponge biology allows for further alternative strategies to obtain DNA or biological material. The specimen of *E. muelleri* was grown from a gemmule (a clonal structure for dispersion) under sterile conditions (Kenny *et al*. 2020). *T. wilhelma* also has a form of clonal reproduction called budding. Sampling a bud is thus an easy way of sampling the sponge without harming it.

For most of the assemblies, DNA isolated from the sponges was sufficient. However, in case of single larvae (*O. pearsei*) and cells (*S. carteri* and *X. testudinaria*), WGAs were performed prior to sequencing. Whether to have sequencing reads derived from (1) a single individual as compared to (2) several individuals or (3) WGA DNA matters as differences due to individual variation and amplification errors can lead to more fragmented assemblies. However, these strategies are a trade-off to avoid overwhelming microbial contamination in the sequencing reads (Fig. 2b). One of the crucial aspects of sponge biology is their pervasive association with microbial symbionts (prokaryotes: bacteria, and archaea), especially in their sessile adult stage (Vacelet 1975; Leys *et al*. 2018). Recognizing this association has led to the classification of sponge species as HMA or LMA sponges. Typically, HMA also implies high microbial diversity and vice versa. Larvae also contain microbial symbionts, albeit to a greatly reduced extent (Björk *et al*. 2019). These microbial symbionts affect sponge genome sequencing in several ways. Extracting DNA from sponges inevitably leads to contamination with microbial DNA. This can be a challenge in the assembly process and lead to contamination and fragmentation in the resulting genome. Identification of microbial sequences can be difficult as there is a lack of both bona fide sponge sequences in databases as well as genomes of deep-sea microbes. At the same time, a locus with similarity to microbial sequences can also originate from horizontal gene transfer (HGT), which was previously shown in the *A. queenslandica* genome (Conaco *et al*. 2016). Conversely, bacterial genes coding for eukaryote-like proteins, present in sponge microsymbionts (Reynolds and Thomas 2016), could potentially be mistaken for true sponge genes. This means that in silico decontamination is challenging. Overall, the most successful contiguous assemblies have so far leveraged Hi-C (*C. reniformis*, *P. ficiformis*, and *E. muelleri*) and/or some form of (synthetic) long reads (*G. barretti*, *O. minuta*, and *T. wilhelma*).

Terpene BGCs have been lately discovered in several genomes of octocoral species (Burkhardt *et al*. 2022; Scesa *et al*. 2022) and sponges (Wilson *et al*. 2023). We therefore analyzed all 13 genomes with antiSMASH to identify possible BGCs: results ranged from 1–2 BGCs (*A. queenslandica* and *C. reniformis*) to 133 BGCs (*X. testudinaria*) (Table 3). While we did not determine whether these gene clusters originate from contamination or genuine cases of HGT, they highlight once more the close association of the sponges with their microbes and raise the possibility that BGCs are relatively widespread in sponge genomes. Interestingly, 1–10 BGCs were still found in chromosome-level assemblies (*C. reniformis*, *P. ficiformis*, and *E. muelleri*), which hints at genuine cases of BGC transferred to the sponge, deserved to be further studied. In the *G. barretti* genome, 21 BGCs were detected (Supplementary Table 1): 1 arylpolyene, 1 betalactone, 1 nonribosomal peptide synthetase (NRPS), 5 NRPS-like, and 13 terpene clusters. This is the third highest number only exceeded by *L. baikalensis* with 69 and *X. testudinaria* with 133 BGCs (Table 2). A full list of all BGCs is included in Supplementary Table 1. Overall, there seems to be no correlation between HMA/LMA status and the number of BGCs detected. It is worth noting that the majority of BGCs in *L. baikalensis* and *X. testudinaria* start at the first position of a short contig which indicates that the BGC is likely truncated and/or incomplete. The high numbers of BGCs for these 2 species might thus be an overestimation due to counting the same BGC multiple times.

To conclude, the *G. barretti* genome is the first genome for the Tetractinellida order, the second most speciose order of demosponges with over 1,100 species (de Voogd *et al*. 2023). All 4 deep-sea sponge genomes published so far are glass sponges (class Hexactinellida) (Francis *et al*. 2023; Santini *et al*. 2023; Schultz *et al*. 2023), with 3 of them published after submission of this study; the *G. barretti* genome is the first deep-sea genome of a demosponge. This genome will firmly establish the North Atlantic *G. barretti* as a prominent deep-sea sponge species for future studies, allowing the generation of new hypotheses about multicellularity, immunity, chemistry, and symbiont/cell recognition and interaction, which could be tested in vitro, thanks to the successful *G. barretti* cell line and CRISPR/Cas12a gene-editing system (Hesp *et al*. 2020, 2023).

## Supplementary Material

jkad192_Supplementary_Data

## Data Availability

The data associated with this study are deposited at the European Nucleotide Archive (ENA) at EMBL-EBI under accession number PRJEB58046. Raw reads from PacBio RS II are ERR10902930, and from PacBio Sequel are ERR10857208, ERR10857206, ERR10857204, ERR10857202, and Illumina HiSeq X Ten ERR10857169. Previously published RNAseq data (BioProject: PRJNA603347, SRA: SRS6083072) include 4 individuals from the Norwegian and Barents seas, sequenced with ScriptSeq V2 (ROV6_3, trawl_5, trawl_6, and trawl_8) and 3 individuals from Sweden sequenced with TruSeq v2 [Geodia_01 (UPSZMC 184975), Geodia_02 (UPSZMC 184976), and Geodia_03 (UPSZMC 184977)] (Koutsouveli, Cárdenas, Santodomingo, *et al*. 2020). Supplemental material available at G3 online.
