# Supplementary material for: Whole genome sequence of the deep-sea sponge Geodia barretti (Metazoa, Porifera, Demospongiae)
Source: G3 (Bethesda). 2023 Aug 24;13(10):jkad192. doi: 10.1093/g3journal/jkad192 (PMC10542158; doi:10.1093/g3journal/jkad192)

**Supplementary figures S1–S4**

**Whole genome sequence of the deep-sea sponge *Geodia barretti* (Metazoa, Porifera, Demospongiae)**

Karin Steffen, Estelle Proux-Wéra, Lucile Soler, Allison Churcher, John Sundh, Paco Cárdenas

**Figures S1–S4.** Blobplots of the genome assembly at different stages. For all plots, each circle represents a contig. The size of the circle is proportional to its length and is plotted by GC proportion and coverage with illumine reads. The legend detail in parentheses the number of sequences, their combined length and N50.

**Figures S1** and **S2** depict the inferred taxonomic composition of the contigs in the polished flye assembly, before (i.e. as part of) the decontamination process. **Figure S1** plot shows taxonomy at phylum level, **Figure S2** plot at superkingdom level. **Figures S3** and **S4** depict the inferred taxonomic composition of the contigs in final assembly, after the decontamination process. **Figure S3** plot shows taxonomy at phylum level, **Figure S4** plot at superkingdom level.

S1
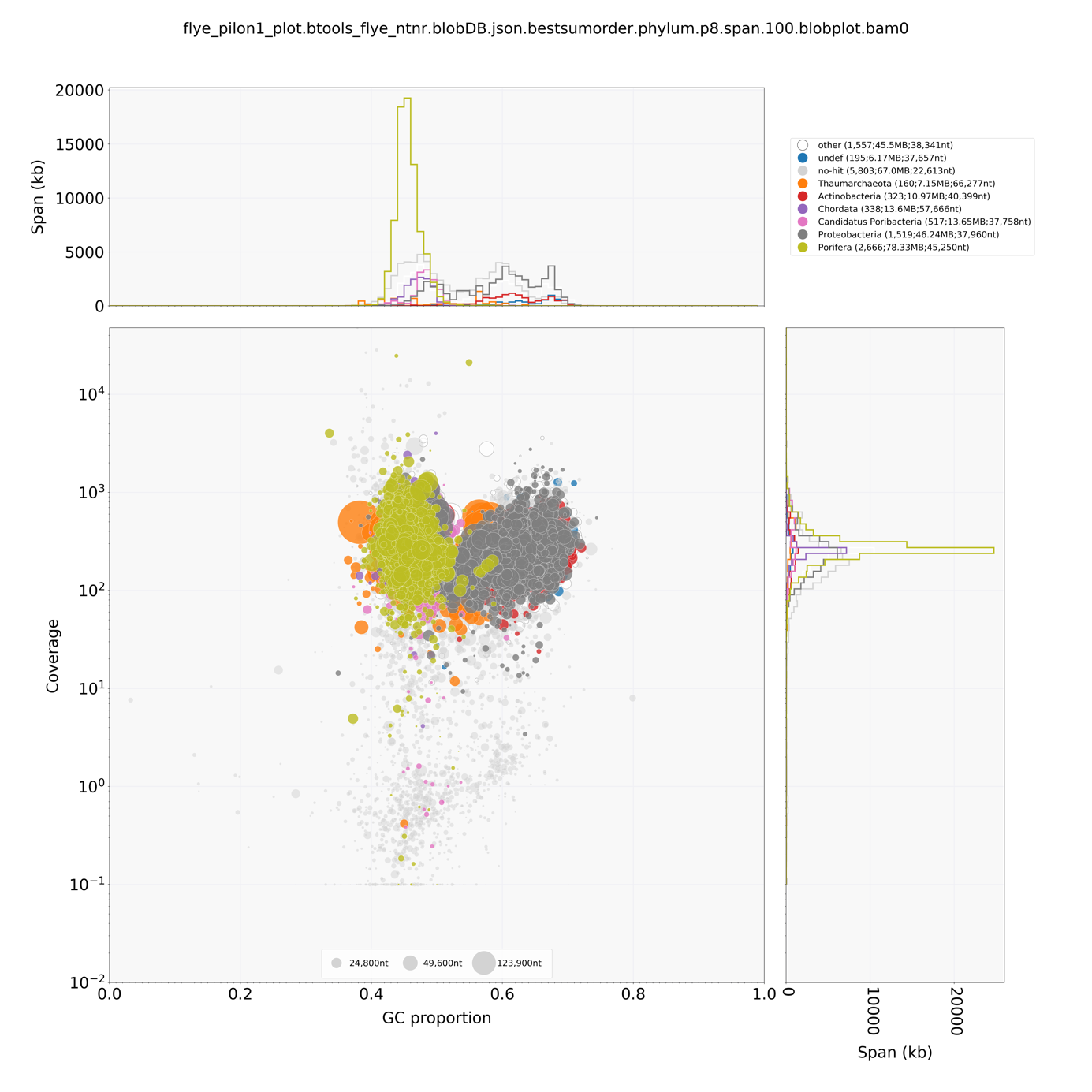
S2
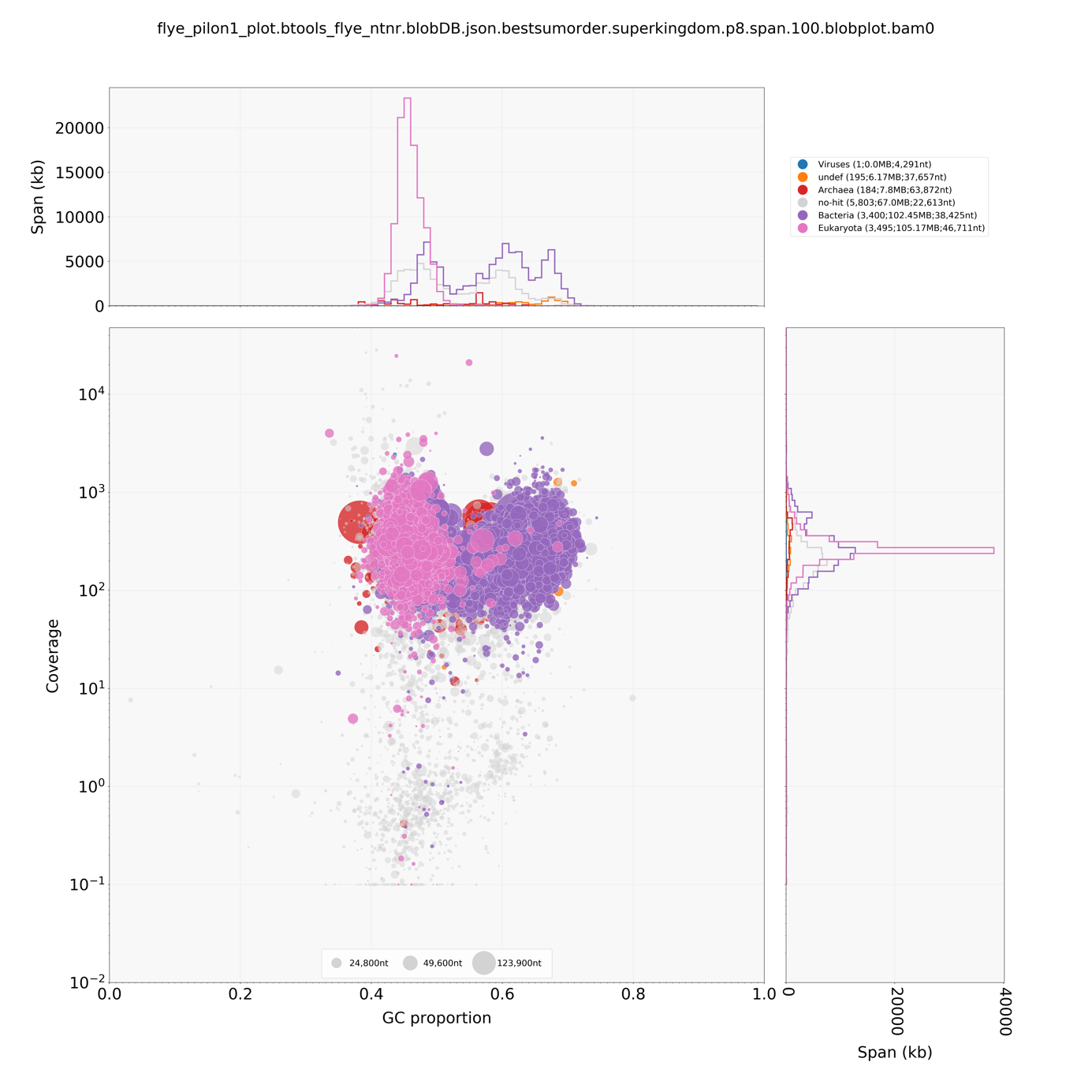


S3
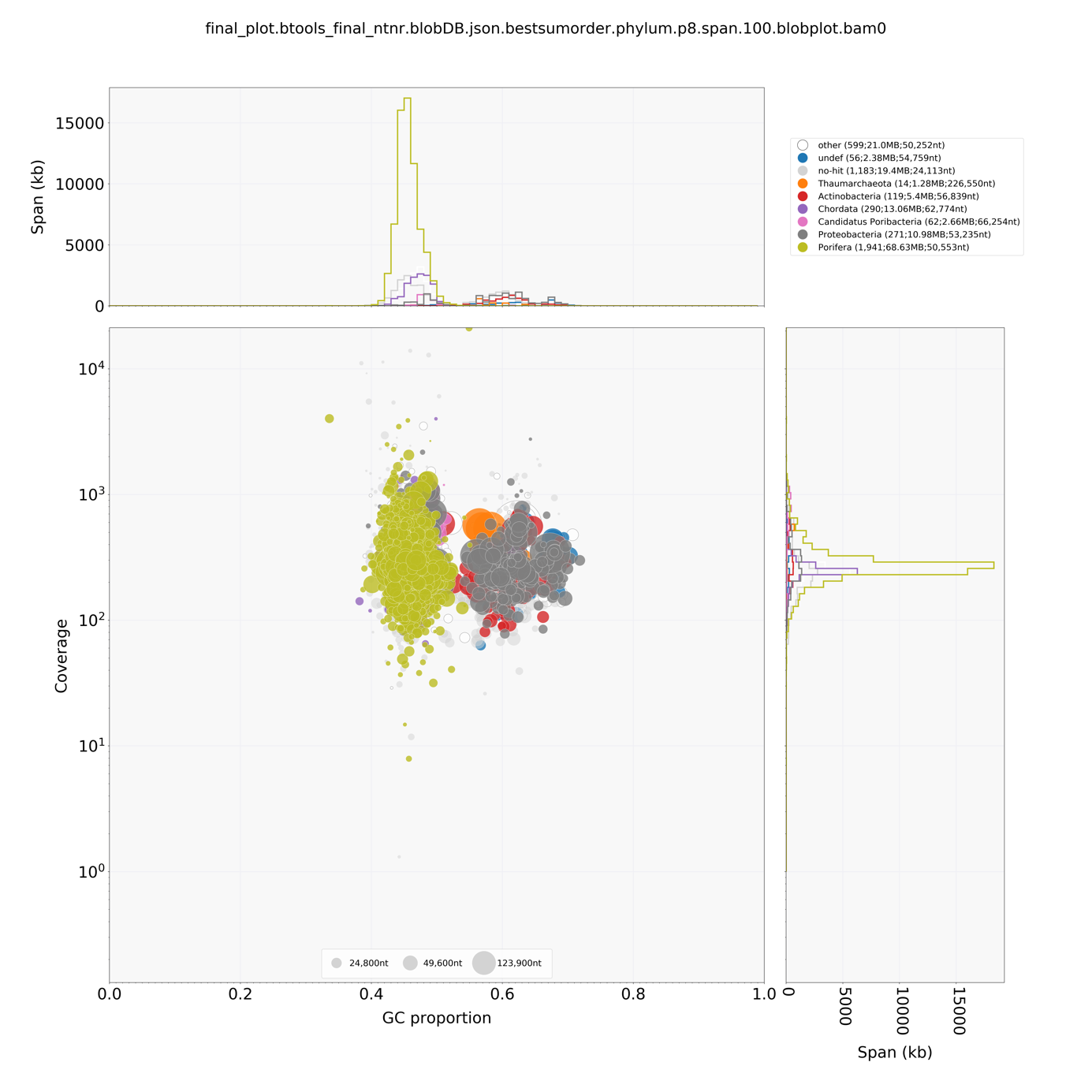
S4
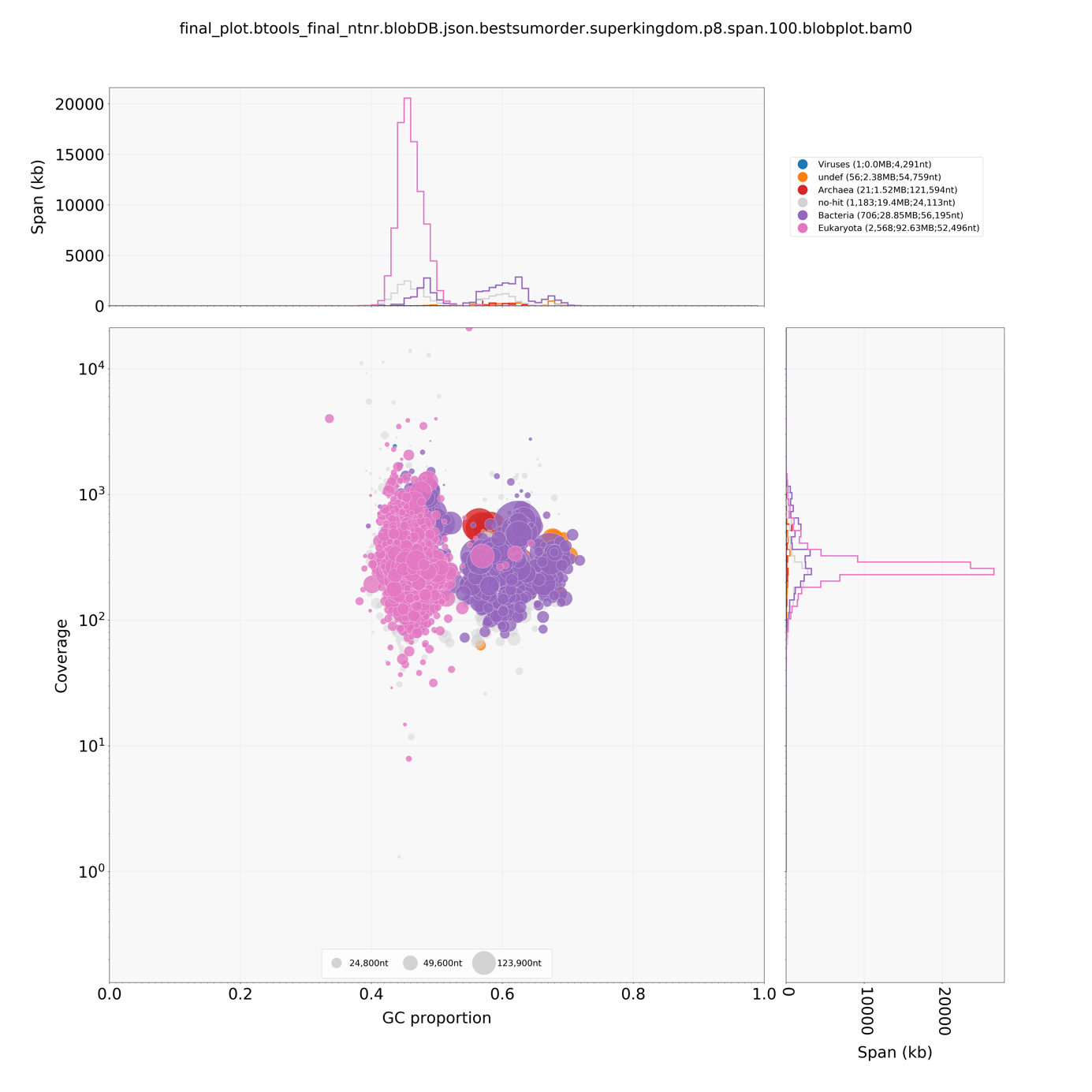

Supplement: jkad192_Supplementary_Data [file jkad192_supplementary_data.zip › Figures_S1-S4_G3-2023-404369.docx]
