# Supplementary material for: Whole genome sequence of the deep-sea sponge Geodia barretti (Metazoa, Porifera, Demospongiae)
Source: G3 (Bethesda). 2023 Aug 24;13(10):jkad192. doi: 10.1093/g3journal/jkad192 (PMC10542158; doi:10.1093/g3journal/jkad192)
Supplement: jkad192_Supplementary_Data [file jkad192_supplementary_data.zip › Table_S1_G3-2023-404369.docx]

**Whole genome sequence of the deep-sea sponge *Geodia barretti* (Metazoa, Porifera, Demospongiae)**

Karin Steffen, Estelle Proux-Wéra, Lucile Soler, Allison Churcher, John Sundh, Paco Cárdenas

**Table S1.** List of all types of BGCs found with antiSMASH in the different sponge genomes published to date.

| BGC_kind | count | species |
| --- | --- | --- |
| terpene | 1 | *Amphimedon queenslandica* |
| terpene | 1 | *Chondrosia reniformis* |
| NRPS | 8 | *Ephydatia muelleri* |
| NRPS,T1PKS | 2 | *Ephydatia muelleri* |
| arylpolyene | 1 | *Geodia barretti* |
| betalactone | 1 | *Geodia barretti* |
| NRPS | 1 | *Geodia barretti* |
| NRPS-like | 5 | *Geodia barretti* |
| terpene | 13 | *Geodia barretti* |
| bacteriocin | 2 | *Halichondria panicea* |
| NRPS-like | 1 | *Halichondria panicea* |
| terpene | 1 | *Halichondria panicea* |
| arylpolyene | 3 | *Lubomirskia baikalensis* |
| bacteriocin | 4 | *Lubomirskia baikalensis* |
| NRPS | 27 | *Lubomirskia baikalensis* |
| NRPS-like | 3 | *Lubomirskia baikalensis* |
| other | 1 | *Lubomirskia baikalensis* |
| resorcinol | 1 | *Lubomirskia baikalensis* |
| T1PKS | 1 | *Lubomirskia baikalensis* |
| T1PKS,NRPS | 1 | *Lubomirskia baikalensis* |
| T1PKS,NRPS-like | 1 | *Lubomirskia baikalensis* |
| T3PKS | 7 | *Lubomirskia baikalensis* |
| terpene | 20 | *Lubomirskia baikalensis* |
| NRPS,T1PKS | 1 | *Oopsacas minuta* |
| terpene | 3 | *Oopsacas minuta* |
| NRPS | 2 | *Oscarella pearsei* |
| phosphonate | 1 | *Oscarella pearsei* |
| NRPS | 2 | *Petrosia ficiformis* |
| NRPS-like | 1 | *Petrosia ficiformis* |
| bacteriocin | 6 | *Stylissa carteri* |
| betalactone | 1 | *Stylissa carteri* |
| NRPS | 3 | *Stylissa carteri* |
| NRPS-like | 1 | *Stylissa carteri* |
| proteusin | 1 | *Stylissa carteri* |
| proteusin,bacteriocin | 1 | *Stylissa carteri* |
| T1PKS | 2 | *Stylissa carteri* |
| bacteriocin | 4 | *Sycon ciliatum* |
| betalactone | 1 | *Sycon ciliatum* |
| hserlactone | 1 | *Sycon ciliatum* |
| lanthipeptide | 1 | *Sycon ciliatum* |
| LAP | 1 | *Sycon ciliatum* |
| siderophore | 1 | *Sycon ciliatum* |
| NRPS | 1 | *Tethya wilhelma* |
| NRPS-like | 1 | *Tethya wilhelma* |
| T1PKS | 1 | *Tethya wilhelma* |
| arylpolyene | 4 | *Xestospongia testudinaria* |
| bacteriocin | 26 | *Xestospongia testudinaria* |
| betalactone | 2 | *Xestospongia testudinaria* |
| ectoine | 5 | *Xestospongia testudinaria* |
| LAP | 1 | *Xestospongia testudinaria* |
| NRPS | 2 | *Xestospongia testudinaria* |
| NRPS-like | 8 | *Xestospongia testudinaria* |
| phosphonate | 3 | *Xestospongia testudinaria* |
| proteusin | 1 | *Xestospongia testudinaria* |
| T1PKS | 20 | *Xestospongia testudinaria* |
| T3PKS | 2 | *Xestospongia testudinaria* |
| terpene | 58 | *Xestospongia testudinaria* |
| transAT-PKS-like | 1 | *Xestospongia testudinaria* |
